# Optical coherence tomography angiography suggests different retinal pathologies in multiple sclerosis and Sjögren’s syndrome

**DOI:** 10.1007/s00415-024-12414-0

**Published:** 2024-05-14

**Authors:** Elisabeth Wolf, Rebecca Wicklein, Lilian Aly, Christoph Schmaderer, Ali Maisam Afzali, Christian Mardin, Thomas Korn, Bernhard Hemmer, Benedikt Hofauer, Benjamin Knier

**Affiliations:** 1 Department of Neurology, Klinikum Rechts Der Isar, TUM School of Medicine and Health, Technical University of Munich, Munich, Germany; 2 Department of Nephrology, Klinikum Rechts Der Isar, TUM School of Medicine and Health, Technical University of Munich, Munich, Germany; 3 Department of Ophthalmology, University Hospital of Erlangen-Nuremberg, Erlangen, Germany; 4 Institute for Experimental Neuroimmunology, TUM School of Medicine and Health, Technical University of Munich, Munich, Germany; 5 Munich Cluster of Systems Neurology (SyNergy), Munich, Germany; 6 Department of Otorhinolaryngology/Head and Neck Surgery, Klinikum Rechts Der Isar, TUM School of Medicine and Health, Technical University of Munich, Munich, Germany; 7 Department of Otorhinolaryngology/Head and Neck Surgery, Tirol Kliniken, Universitätskliniken Innsbruck, University of Innsbruck, Innsbruck, Austria; 8 Department of Neurology, Diakonie-Klinkum Schwäbisch Hall, Schwäbisch Hall, Germany

**Keywords:** Multiple sclerosis (MS), Sjöegren’s syndrome, Optical coherence tomography angiography (OCTA), Differential diagnosis

## Abstract

**Background:**

While retinal vessel changes are evident in the eyes of patients with relapsing–remitting multiple sclerosis (RRMS), changes in the vasculature of possible MS mimics such as primary Sjögren’s syndrome (pSS) remain to be determined. We investigated the potential of retinal optical coherence tomography (OCT) angiography (OCTA) as diagnostic tool to differentiate between patients with RRMS and pSS.

**Methods:**

This cross-sectional study included patients with RRMS (*n* = 36), pSS (*n* = 36) and healthy controls (*n* = 30). Participants underwent clinical examination, assessment of visual acuity, retinal OCT, OCTA, and serum markers of glial and neuronal damage. We investigated the associations between OCTA parameters, visual functions, and serum markers. Eyes with a history of optic neuritis (ON) were excluded from analysis.

**Results:**

We observed a significant thinning of the combined ganglion cell and inner plexiform layer in the eyes of patients with RRMS but not with pSS, when compared to healthy controls. Retinal vessel densities of the superficial vascular complex (SVC) were reduced in both patients with RRMS and pSS. However, retinal vessel rarefication of the deep vascular complex (DVC) was only evident in patients with pSS but not RRMS. Using multivariate regression analysis, we found that DVC vessel loss in pSS patients was associated with worse visual acuity.

**Conclusions:**

Compared to patients with RRMS, rarefication of deep retinal vessels is a unique characteristic of pSS and associated with worse visual function. Assuming a disease-specific retinal vessel pathology, these data are indicative of a differential affliction of the gliovascular complex in the retina of RRMS and pSS patients.

**Supplementary Information:**

The online version contains supplementary material available at 10.1007/s00415-024-12414-0.

## Introduction

Multiple sclerosis (MS) is a chronic autoimmune disorder characterized by inflammation, demyelination, and axonal loss in the central nervous system (CNS). Ocular manifestations, particularly optic neuritis (ON), are commonly observed in individuals with MS, affecting up to 70% of patients during the disease course⁠ [1]. There are multiple diseases with similar ocular involvement acting as potential MS mimics, such as the primary Sjögren’s syndrome (pSS) [2]. PSS is an autoimmune disease characterized by the dysfunction of exocrine glands leading to dryness of the mouth and eyes [3]. The lack of a gold standard for diagnosis and diverse manifestations lead to delayed diagnosis of up to 10 years and recurrent misdiagnosis as MS [4–8]. Neurological involvement is common and its prevalence varies between 10 and 60% with up to 25% extra-glandular ocular manifestations [9, 10].

Optical coherence tomography (OCT) has been used extensively in the past and allows a non-invasive high-resolution visualization of retinal anatomy. Thinning of inner retinal layers including the peripapillary retinal nerve fiber layer (pRNFL) and the combined ganglion cell and inner plexiform layer (GCIP) is consistently observed in patients with MS. GCIP alterations have been associated with neurodegenerative processes of the CNS and are indicative of a worse disease prognosis. In pSS, there is contradictive literature concerning changes of the retinal architecture. Both a lack of alterations [11, 12] and an atrophy of the whole posterior pole or inner retinal layers have been described⁠ [6, 7, 13]. While changes of the retinal architecture can be visualized by OCT, optical coherence tomography angiography (OCTA) complements the original technique by providing high-resolution images of the retinal vasculature based on the changes of reflectivity due to moving blood cells. Changes of retinal vessels in MS have been controversially discussed [14]; however, most studies show a reduction of the superficial vascular complex (SVC), while the DVC remains unchanged [15–17]. OCTA data in patients with pSS are rare. Small cohort studies reported a reduction of the superficial (SVC) and deep (DVC) vascular complex in comparison to healthy individuals [12, 13, 18].

Since early detection and management of ocular complications in MS and pSS are crucial for preventing irreversible damage, this study aimed to investigate the differences between pSS and MS patients without history of optic neuritis (ON) in OCTA studies. Furthermore, we searched for the associations of retinal vessel changes and alterations of the retinal architecture, visual function, and markers of neuronal damage generating novel hypotheses of the underlying pathology.

## Methods

### Study design

This cross-sectional cohort study included 36 patients with pSS and 36 sex- and age-matched patients with RRMS as well as 30 healthy controls (HC). Patients with pSS were recruited at the Department of Neurology and the Department of Otorhinolaryngology/Head and Neck Surgery, Klinikum rechts der Isar, TUM School of Medicine and Health, between 2020 and 2022. PSS diagnosis followed the 2016 ACR-EULAR protocol [19], a classification system based on five items (antibody positivity, Schirmer’s test, ocular staining, salivary flow rate, and lymphocytic sialadenitis). Patients with secondary Sjögren’s syndrome were not enrolled in this study. Age- and sex-matched RRMS patients with a comparable portion of an ON history were retrospectively identified from an ongoing observational cohort study at the Department of Neurology on the natural course of RRMS (TUM-MS). RRMS diagnostic criteria were retrospectively revised according to the 2017 McDonald consensus criteria [20]. Age- and sex-matched controls were recruited from healthy volunteers at the Department of Neurology. The primary objective of this study was to characterize the alterations of the retinal vasculature in RRMS and pSS, and to evaluate the capacity of OCTA to differentiate between RRMS and pSS. As secondary objectives, we investigated associations of the retinal vasculature and visual acuity, disability, and soluble markers associated with tissue damage. All participants underwent retinal OCT and OCTA examination, a thorough neurological check-up with assessment of the Expanded Disability Status Scale (EDSS), evaluation of the high-contrast (HCVA) and low-contrast visual acuity (LCVA), and blood sampling for analysis of serum levels of the neurofilament light chain (NfL) and of the glial fibrillary acidic protein (GFAP). We took an in-depth medical history from all individuals, particularly as to a history of former ON.

We excluded subjects with a substantial eye disease affecting the retinal architecture or vasculature like diabetes, and refractory errors of more than 6 diopters. Eyes with insufficient OCT/OCTA quality were removed from our study.

### Standard protocol approvals, registration, patient consent, and data availability

This study met STROBE guidelines [21], was approved by the ethics commission of the Technical University of Munich School of Medicine (166/16S, 2023-526-S-KH, 9/15 s), and complied with the Declaration of Helsinki. All participants and patients provided written informed consent. The data are not publicly available due to privacy or ethical restrictions. Data can be shared in an anonymized way upon reasonable request by any qualified investigator.

### OCT and OCTA analysis

Conventional OCT images, including examination of pRNFL and a 30° × 25° macular scan, were acquired as previously described [22]. To ensure adequate quality control, all images were checked according to the OSCAR-IB criteria [23]. Retinal layer segmentation was performed automatically by an in-built software algorithm (Eye Explorer, v2.5.4) and manually corrected if needed.

OCTA data were recorded by a spectral-domain OCT with angiography module (Heidelberg Engineering Spectralis OCT2) under low-lighting conditions on both eyes of each individual, as previously summarized [15]. During examination, en face images and decorrelation signals were measured within a 2.9 × 2.9 mm region of interest, directly focusing on the fovea centralis. An active eye-tracking algorithm was incorporated to minimize motion artifacts. Vessel densities of the superficial (SVC) and deep (DVC) vascular complexes were assessed within a circle around the fovea between 0.8 mm and 2.9 mm eccentricity (area 6.1 mm^2^) using the Erlangen Angio tool [24]. A MatLab algorithm (MathWorks, vR2019b) was employed to quantify the foveal avascular zone (FAZ), as previously outlined [15]. To achieve maximum reliability, OCTA images underwent strict quality control according to OSCAR-MP criteria [25]. Accordingly, OCTA images with obvious problems, insufficient signal strength of Q < 30, centration errors, segmentation algorithm failure, retinal pathologies, motion artifacts affecting more than 25% of the image area, and projection artifacts were excluded. Subclinical ON was assumed when the inter-eye difference of pRNFL and GCIP values as measured by OCT exceeded 5 and 4 µm, respectively [26].

### Assessment of the visual function

Monocular visual testing included high-contrast (100%) and low-contrast (2.5%) visual acuity. Individuals were asked for the reproduction of alphabetic characters using retro-illuminated Early Treatment Diabetic Retinopathy Study charts (EDTRS) at a two-meter distance. Visual measurement was performed in best-corrected refraction, and a minimum of three characters in the smallest read line was necessary to define visual acuity as the decimal of the Snellen fraction.

### Analysis of blood samples

Serum specimens were stored at − 80 degrees in the Biobank of the Department of Neurology (Joint Biobank Munich in the framework of the German Biobank Node) until final use. Concentrations of serum NfL and GFAP were calculated using ultrasensitive single-molecule array (Simoa) technology on an HD-X analyzer (Simoa, Quanterix, NF light Simoa Assay Advantage Kit, GFAP Simoa Discovery Kit). Samples were processed according to the manufacturer’s instructions. Samples exceeding an intra-assay coefficient of variation (CV) of 10% were excluded. Age-dependent z-score for Nfl was generated, as previously described [27].

### Statistical analysis

Statistical analyses were performed using GraphPad Prism (v9.3.1). To account for inter-eye correlations, we applied the paired eye statistical approach [28]. Mean values of both eyes were used as one data point if data of both eyes were available. To evaluate the quantitative differences between two groups, we used the unpaired t test if data were normally distributed and a non-parametric Mann–Whitney U test if not. We performed an ordinary one-way analysis of variance (ANOVA) with Tukey’s multiple comparisons or a non-parametric Kruskal–Wallis test with Dunn’s multiple comparisons to outline statistical differences of three groups (MS, pSS, and HC). We used multiple linear regression models, corrected for age and sex, to evaluate the associations between OCT or OCTA values on disease patterns, markers for tissue damage, and visual acuity. Data are provided as median (25–75% interquartile range [IQR]) and respective estimates (ß-value) as regression parameters. An alpha of < 0.05 was accepted as significant.

## Results

### Study cohort characteristics

In this cross-sectional study, we enrolled 36 patients with pSS, 36 age- and sex-matched RRMS patients, and 30 HC. As depicted in Table 1, ages were comparable across all groups, and 97% of all participants were female. No patient with pSS and RRMS suffered from a clinical ON in the past. Patients with pSS had a higher EDSS than individuals with RRMS and a worse visual acuity as compared to HC. Besides diminished visual acuity, sensory impairment was the predominant neurological manifestation within the pSS cohort, while sensory and motor function was primarily affected in patients with RRMS (see Table 2). Subclinical ON was found in one pSS and three RRMS patients. We found comparable serum levels and z-scores of NfL and GFAP across all three cohorts (Table 1).

**Table 1:** Demographics

	RRMS (*n* = 36)	pSS (*n* = 36)	HC (*n* = 30)	*p*-value
Female, no. (%)	35 (97.2)	35 (97.2)	29 (96.7)	0.99
Age, years	54 (46–58)	55 (48–61)	51 (43–56)	0.12
Disease duration, years	11 (7–14)	16 (7–26)	n.a	0.07
EDSS score	1.8 (1.0–2.0)	2.5 (2.0–3.4)	n.a	** < 0.0001**
Immunotherapy (IT) no (%)	27 (75.0)	21 (58.3)	n.a	0.21
- more than 1 IT	0 (0)	5 (24)	n.a	
- Azathioprine	0 (0)	2 (9.5)	n.a	
- Metothrexate	0 (0)	3 (14.3)	n.a	
- Glucocorticoids	0 (0)	6 (28.6)	n.a	
- Hydroxychloroquine	0 (0)	10 (47.6)	n.a	
- Rituximab	0 (0)	3 (14.3)	n.a	
- Ocrelizumab	3 (11.1)	0 (0)	n.a	
- Interferon beta	6 (20.0)	0 (0)	n.a	
- Fingolimod	6 (20.0)	0 (0)	n.a	
- Glatirameracetate	5 (18.5)	0 (0)	n.a	
- Dimethyl furamate	4 (14.8)	0 (0)	n.a	
- others	3 (11.1)	3 (14.3)	n.a	
HCVA NON	1.0 (0.7–1.1)	0.8 (0.7–0.9)	0.9 (0.7–1.1)	**0.02**^a^
LCVA NON	0.3 (0.2–0.4)	0.2 (0.2–0.3)	0.3 (0.2–0.4)	**0.04**^b^
NfL (pg/mL)^c^	10.9 (6.9–14.2)	9.7 (7.0–13.0)	10.4 (7.9–13.0)	0.71
NfL (z-score)^c^	0.3 (− 0.4–0.8)	0.2 (− 0.4–1.1)	0.7 (0.3–1.2)	0.52
GFAP (pg/mL)^d^	113 (82–129)	94 (72–122)	110 (78–135)	0.67

Values are provided as median (25–75% interquartile range). Kruskal–Wallis test was used to analyze age, sex, HCVA, LCVA, NfL and GFAP and the Mann–Whitney-U test for disease duration, EDSS and IT. Ordinary one-way ANOVA was performed for NfL z-score analysis

*EDSS* Expanded disability status scale, *GFAP* glial fibrillary acidic protein, *HC* healthy control, *HCVA* high-contrast visual acuity, *IT* immunotherapy, *NfL* neurofilament light chain, *pSS* primary Sjögren‘s syndrome, *RRMS* relapsing–remitting multiple sclerosis, *LCVA* low-contrast visual acuity

^a^HCVA: *p*_HC,MS_ > 0.99; *p*_HC,pSS_ = 0.10

^b^LCVA: *p*_HC,MS_ > 0.99; *p*_HC,pSS_ = 0.12

^c^n_RRMS_ = 27, n_pSS_ = 15, n_HC_ = 23

^d^n_RRMS_ = 25, n_pSS_ = 15, n_HC_ = 23

**Table 2:** Neurological impairment in patients with pSS and RRMS

	pSS *n* = 36	RRMS *n* = 36
Visual function, no. (%)	32 (89)	23 (64)
Brainstem function, no. (%)	19 (53)	4 (11)
Muscle and motor function, no. (%)	12 (33)	19 (53)
Cerebellar function, no. (%)	10 (28)	11 (31)
Sensory system, no. (%)	31 (86)	20 (56)
Bowel and bladder function, no. (%)	14 (39)	6 (17)
Cognitive/cerebral function, no. (%)	6 (17)	9 (25)

relapsing–remitting multiple sclerosis (MS), primary Sjögren‘s syndrome (pSS)

### Alterations of the retinal architecture and vasculature in RRMS and pSS

**Fig. 1:**
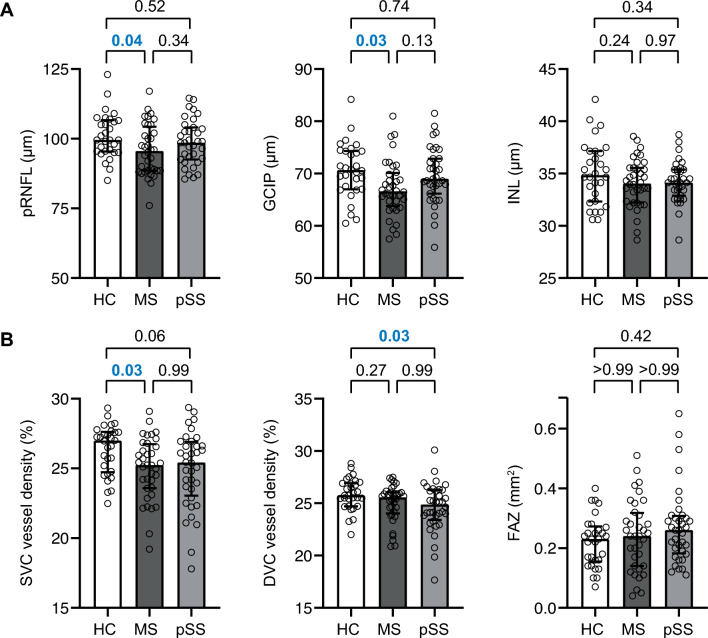
Changes of the retinal architecture and vasculature in RRMS and pSS. **A** pRNFL, GCIP and INL in HC, RRMS and pSS as calculated by one-way ANOVA B) SVC, DVC and FAZ in HC, RRMS and pSS as calculated by Kruskal–Wallis test. (A + B) Data are shown as median (25–75% interquartile range), symbols show individual patient values. *DVC* deep vascular complex, *FAZ* foveal avascular zone, *GCIP* ganglion cell and inner plexiform layer, *HC* healthy control, *INL* inner nuclear layer, *MS* multiple sclerosis, *pRNFL* peripapillary retinal nerve fiber layer, *pSS* primary Sjögren‘s syndrome, *RRMS* relapsing remitting multiple sclerosis, *SVC* superficial vascular complex

We excluded 3 of 204 eyes from OCT (RRMS 1, pSS 2, HC 0) and 38 of 204 eyes from OCTA analysis (RRMS 16, pSS 15, HC 7) due to poor image quality. In the first step, we applied OCT analysis to search for alterations of the retinal architecture across all three groups primarily in eyes without a subclinical ON (sON) history. As expected [29], patients with RRMS but not pSS revealed thinning of the pRNFL and GCIP as compared to HC in eyes without a sON history (Fig. 1A). There were no detectable changes in retinal layer thicknesses between RRMS and pSS. Thickness measures of deeper retinal layers were comparable across all groups (data not shown).

In the second step, we used OCTA to evaluate changes of the retinal vasculature. In our study, vessel densities of the SVC were reduced in patients with RRMS and by trend in pSS as compared to HC irrespective of a history of ON (Fig. 1B). In patients with pSS without sON but not RRMS we observed a moderate rarefication of vessel structures within in DVC as compared to healthy individuals. We did not observe an alteration of the FAZ in any cohort. Ten patients diagnosed with pSS were currently undergoing hydroxychloroquine therapy for an average duration of 6 years. Despite a reduced inner nuclear layer (INL) in patients with hydroxychloroquine, we did not see any differences in OCT or OCTA measures between patients receiving hydroxychloroquine and those who were not (see supplemental Table 2). We did not recognize any differences in both OCT and OCTA measures when comparing eyes of RRMS and pSS patients and a possible history of sON (see supplemental Table).

### Association of the retinal vasculature, disability, and neurodegeneration

**Fig. 2:**
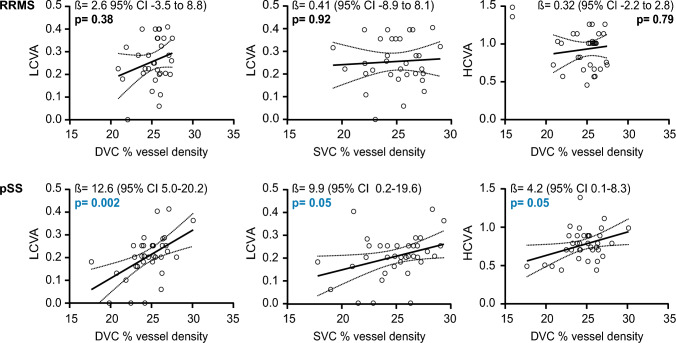
Association of retinal architecture and vasculature with visual acuity in RRMS and pSS patients. Correlation of LCVA with DVC and SVC measures as well as correlation of HCVA with DVC measures in patients with pSS (upper panel) and RRMS (lower panel). β regression estimates and 95% confidence interval (CI) corrected for age and sex; symbols show individual patient values. *DVC* deep vascular complex, *HC* healthy control, *HCVA* high-contrast visual acuity, *LCVA* low-contrast visual acuity, *pRNFL* peripapillary retinal nerve fiber layer, *pSS* primary Sjögren‘s syndrome, *RRMS* relapsing–remitting multiple sclerosis, *SVC* superficial vascular complex

In the last step, we searched for the associations between retinal vessel densities, visual disability, and serum markers of glial and neuronal damage. Focusing on visual acuity, SVC and DVC measures in patients with pSS but not RRMS showed a significant correlation with low-contrast visual acuity (LCVA). The DVC was also associated with worse HCVA in pSS only (Fig. 2). There were no associations of EDSS or neurodegenerative markers with OCT or OCTA measures.

## Discussion

In the current study, we found distinct changes in the retinal architecture and vasculature in patients with RRMS and pSS. Individuals with RRMS revealed atrophy of inner retinal layers (pRNFL, GCIP) and vessel rarefication within the SVC whereas vessel loss within both the SVC and DVC was evident in patients with pSS irrespective of changes in the retinal architecture. Our findings are consistent with the idea of a disease-specific retinal vessel pathology in RRMS versus pSS.

Prior studies on changes in the retinal thicknesses in pSS are controversial [6, 7, 11, 12, 30]. As has been reported [11, 12], we did not see changes in the average retinal layer thicknesses in patients with pSS. However, a reduction of the total macular RNFL⁠ [6, 30] and the whole posterior pole [7] have been described. Further, antibody positivity, especially of anti-Sjögren’s syndrome type B (anti-SS-B) antibodies, was shown to be associated with inner retinal layer atrophy [6, 30] but was not measured in our study. As expected, we found a reduction of the SVC in patients with RRMS [15–17] and pSS [13]. The loss of deep retinal vessels, however, was only visible in patients with pSS and not in patients with RRMS. This findings are coherent with data from smaller pSS cohorts [12, 13, 18]. In our study, the vessel loss of both SVC and DVC in pSS was linked to low visual acuity which has not been described previously. In contrast to vision loss in RRMS patients [31], reduced visual acuity was not associated with inner retinal layer thinning in pSS suggesting a different underlying pathophysiology.

As shown by others [18], we did not find significant changes in the size of the FAZ. However, alterations in the retinal microvasculature have been previously linked to an increased size and reduced circularity of the FAZ in diabetic retinal ischemia in particular of the deep vessels [32]. In other connective tissue diseases like systemic lupus erythematosus, an increase in FAZ sizes is controversially discussed, but often assumed in connection with retinal ischemia [33]. Assuming a similar phenotype in pSS as in other connective tissue diseases, an increase in FAZ area should be investigated with a more sensitive approach using longitudinal intra-individual comparisons.

Studies have shown a dosage-dependent atrophy of inner retinal layers and the retina pigment epithelium due to hydroxychloroquine [34] while others did not find any layer differences [7, 11]. By applying OCTA, a small cohort study found retinal vessel atrophy in some sectors of the deep vascular complex and in the superior sector of the superficial complex in pSS patients under hydroxychloroquine treatment [35] while others could not find any differences [18]. Further, patients with various autoimmune diseases showed an increase in FAZ as well as decreased para-/perifoveal vessel densities in patients taking hydroxychloroquine for more than 5 years [36] while others saw an increase in vessel densities [37]. Despite the exclusion of patients under hydroxychloroquine, pSS patients still exhibited a reduced vessel density [12] like our cohort which is why we assume a hydroxychloroquine-independent effect.

We did not see any differences in serum NfL or GFAP in neither pSS nor RRMS in comparison to healthy control. In pSS, studies have shown normal NfL levels during remission and an association of NfL with active disease [38, 39]. Since the pSS patients in our cohort did not show signs of active disease, normal serum NfL levels in our study were consistent with prior reports. In contrast, elevated NfL levels in RRMS are indicative of disease activity and have demonstrated responsiveness to immunotherapy [40, 41]. Notably, RRMS exhibits a less pronounced age-related increase in NfL levels compared to healthy individuals and other neurodegenerative diseases [42]. Compared to typical RRMS studies, our cohort, with an advanced median age of 54 years, demonstrated a relatively benign disease course (median EDSS 1.8). Additionally, participating patients reported neither recent relapses nor relapse-independent progression and 75% received immunotherapy. Similar results have been found for GFAP levels in RRMS [43]. Considering this, our findings seem in line with the literature.

Different hypotheses have been proposed to explain the pathophysiological mechanisms behind superficial retinal vessel loss in MS. These include primary and secondary effects of either altered metabolic states of retinal cells or relapse-independent inflammatory processes. Firstly, inner retinal layers, like the GCIP and the RNFL, are supplied with oxygen by the SVC [44]. Acute ON [15] and retrograde axonal degeneration due to inflammation in the visual pathway [45, 46] lead to atrophy of inner retinal layers. The reduced oxygen demand might consecutively cause a rarefication or decreased perfusion of superficial vessels. Secondly, research in our group has linked inflammatory processes in the CNS to ON-independent superficial vessel loss [47]. Therefore, we suspect that inflammation-related mechanisms might be responsible for triggering significant alterations in the retinal microvasculature independent of relapses.

The pathophysiology behind pSS is complex and not fully understood [48, 49]. It involves the activation of cytokines like interferons, leading to B- and later T-cell activation and infiltration in particular of exocrine glands. It has been shown in the past that vision-threatening ocular involvement in pSS is associated with systemic disease manifestations like nephritis, peripheral neuropathy and vasculitis [9]. There are no data on retinal histology in ocular manifestations in pSS; however, nerve biopsies in peripheral neuropathy have found evidence of perivascular inflammatory infiltrates and other vessel abnormalities including (necrotizing) vasculitis [50]. Further it has been shown that anti-Sjögren’s syndrome type A (anti-SSA) antibodies are associated with vasculitis [51], in particular retinal vasculitis, as shown in a case report based on fluorescence angiography [52], and are more commonly observed in central nervous system manifestations [53]. In our study, we observed a loss of both superficial and deep retinal vessels without significant alterations of the retinal layer architecture itself. We also saw an association of vascular changes and not layer atrophy with impaired visual function. Taking this into account, we suspect a primarily vascular pathology in patients with pSS.

Our study has several limitations. Our samples sizes are limited, and we conducted an exploratory analysis without replication in a second. Therefore, we cannot rule out any false positive results. Especially pSS is a rather rare disease and larger cohorts have not been published so far. Furthermore, our study contains only cross-sectional data. A longitudinal study with a larger sample size is necessary to determine changes during the disease course. Additionally, neurological assessments of patients with pSS relied solely on comprehensive clinical examinations, lacking supplementary electrophysiological diagnostics or imaging. Consequently, a differentiated perspective on pSS patients with central in contrast to peripheral nervous system involvement is not provided and should be the subject of further studies. Differential diagnosis takes place in early stages of disease therefore data on differences during disease onset or early ocular manifestations are necessary. In accordance with the female predominance of pSS, this study only provides limited data on findings in male patients with pSS. A large male cohort would be necessary to evaluate sex differences in OCT and OCTA measures in pSS. Further, pre-existing conditions affecting the visual system like diabetes were gathered through a comprehensive medical history only. An impact of undiscovered comorbidities on our data cannot be ruled out. Moreover, we were not able to include MRI findings or further instrumental diagnostic procedures like visual evoked potentials, visual field testing or electroneurography into our analysis. Also, we did not use the EULAR Sjögren Syndrome Patient Reported Index (ESSDAI) [54] to quantify disease burden which impairs comparisons with other studies involving pSS. However, the ESSDAI comprises only two neurological of 12 items: CNS, including ocular involvement, and peripheral nervous system. Therefore, we chose the EDSS to facilitate the comparison with MS patients and better captivate the neurological burden of disease. Furthermore, OCTA is extremely susceptible to imaging artifacts, in particular in patients with impaired vision. In this study, OCTA examinations were conducted by experienced technicians and underwent rigorous quality control [25]. OCTA only provides data on vessel perfusion and not morphology. Therefore, concerning reduced vessel densities, we cannot differentiate between true vessel loss, wall thickening or constriction.

In conclusion, a distinct atrophy of retinal vessels can be observed during MS and pSS suggesting different underlying disease mechanisms. After validation in larger, longitudinal cohorts, OCTA might allow for differential diagnosis of RRMS and pSS.

### Supplementary Information

Below is the link to the electronic supplementary material.

Supplementary file1 (DOCX 15 KB)

Supplementary file2 (DOCX 14 KB)
