# Supplementary material for: Optical coherence tomography angiography suggests different retinal pathologies in multiple sclerosis and Sjögren’s syndrome
Source: J Neurol. 2024 May 14;271(7):4610–9. doi: 10.1007/s00415-024-12414-0 (PMC11233347; doi:10.1007/s00415-024-12414-0)
Supplement: Supplementary file 1 — Supplementary file1 (DOCX 15 KB) [file 415_2024_12414_MOESM1_ESM.docx]

**Supplemental Table 1: Subclinical Optic Neuritis in pSS and RRMS**

|  | NON RRMS  n=33 | NON pSS  n=35 | NON*  p-value | sON RRMS  n=3 | sON pSS  n=1 | ON*  p-value |
| --- | --- | --- | --- | --- | --- | --- |
| SCV (% VD) | 25.4 (23.6-26.8) | 25.4 (22.9-26.9) | 0.89 | 25.0 (23.1-26.1) | 25.4 (25.4-25.4) | 0.74 |
| DVC (% VD) | 25.4 (24.1-26.0) | 25.1 (23.3-26.3) | 0.37** | 25.9 (22.1-26.5) | 24.0 (24.0-24.0) | 0.80 |
| FAZ (mm²) | 0.2 (0.1-0.3) | 0.3 (0.2-0.3) | 0.52** | 0.2 (0.1-0.3) | 0.3 (0.3-0.3) | 0.50 |
| pRNFL (µm) | 96.0 (88.5-103.5) | 98.5 (92.3-103.6) | 0.31 | 89.0 (76.0-105.5) | 105.0 (105.0-105.0) | 0.47 |
| GCIP (µm) | 66.7 (64.1-70.8) | 68.8 (65.9-73.3) | 0.10 | 64.7 (63.0-66.9) | 69.3 (69.3-69.3) | 0.18 |
| INL (µm) | 34.0 (32.3-35.5) | 34.1 (33.3-35.4) | 0.80 | 35.0 (28.7-36.8) | 32.2 (32.2-32.2) | 0.82 |

* Unpaired t-test if not stated otherwise ** Mann-Whitney test

Abbreviations: deep vascular complex (DVC), foveal avascular zone (FAZ), combined ganglion cell and inner plexiform layer (GCIP), inner nuclear layer (INL), non-optic neuritis (NON), peripapillary retinal nerve fibre layer (pRNFL), primary Sjögren‘s syndrome (pSS), relapsing-remitting multiple sclerosis (RRMS), subclinical optic neuritis (sON), superficial vascular complex (SVC), vessel density (VD)
