# Supplementary material for: Optical coherence tomography angiography suggests different retinal pathologies in multiple sclerosis and Sjögren’s syndrome
Source: J Neurol. 2024 May 14;271(7):4610–9. doi: 10.1007/s00415-024-12414-0 (PMC11233347; doi:10.1007/s00415-024-12414-0)
Supplement: Supplementary file 2 — Supplementary file2 (DOCX 14 KB) [file 415_2024_12414_MOESM2_ESM.docx]

**Supplemental Table 2: Hydroxychloroquine treatment in patients with pSS**

|  | **Non-HCQ (n=16)** | **HCQ (n=10)** | **p-value** |
| --- | --- | --- | --- |
| mean time of intake (years) |  | 6 |  |
| SCV (% vessel density) | 26.0 (22.5-26.8) | 24.8 (23.5-27.0) | 0.95 |
| DVC (% vessel density) | 24.9 (23.7-26.2) | 24.8 (23.0-27.0) | 0.56 |
| FAZ (mm²) | 0.2 (0.2-0.3) | 0.3 (0.2-0.3) | 0.92 |
| pRNFL (µm) | 99.0 (92.5-104.5) | 98.0 (92.3-104.1) | 0.90 |
| GCIPL (µm) | 69.3 (66.4-74.8) | 68.7 (65.8-71.4) | 0.45 |
| INL (µm) | 34.5 (33.6-35.5) | 33.0 (31.9-34.4) | **0.01** |

Abbreviations: hydroxychloroquine (HCQ), primary Sjögren‘s syndrome (pSS)
